# The Membrane Transporter OAT7 (SLC22A9) Is Not a Susceptibility Factor for Osteoporosis in Europeans

**DOI:** 10.3389/fendo.2020.00532

**Published:** 2020-08-18

**Authors:** Anne T. Nies, Stefan Weiss, Elke Schaeffeler, Anke Hannemann, Uwe Völker, Henri Wallaschofski, Matthias Schwab

**Affiliations:** ^1^Dr. Margarete Fischer-Bosch Institute of Clinical Pharmacology, Stuttgart, Germany; ^2^University of Tübingen, Tübingen, Germany; ^3^Interfaculty Institute for Genetics and Functional Genomics, University Medicine Greifswald and University of Greifswald, Greifswald, Germany; ^4^DZHK (German Centre for Cardiovascular Research), Partner Site Greifswald, Greifswald, Germany; ^5^Institute of Clinical Chemistry and Laboratory Medicine, University Medicine Greifswald, Greifswald, Germany; ^6^Departments of Clinical Pharmacology, Pharmacy and Biochemistry, University of Tübingen, Tübingen, Germany

**Keywords:** estrone sulfate transporter, SLC22A9, osteoporosis, human association studies, sex steroids

## Abstract

Bone production, maintenance, and modeling are a well-balanced process involving mineralization by osteoblasts and resorption by osteoclasts. Sex steroid hormones, including their conjugated forms, contribute majorly to maintaining this balance. Recently, variants in the *SLC22A9* gene have been associated with osteoporosis in Korean females. We had recently shown that *SLC22A9*, encoding organic anion transporter 7 (OAT7), is an uptake transporter of estrone sulfate and identified several genetic variants in Europeans leading to functional consequences *in vitro*. We therefore hypothesized that *SLC22A9* genetic variants may contribute to the pathophysiology of osteoporosis in Europeans. To test this hypothesis, we examined the associations of *SLC22A9* variants with bone quality, fractures, and bone turnover markers. We genotyped *SLC22A9* variants in 5,701 (2,930 female) subjects (age range, 20–93 years) extracted from the population-based Study of Health in Pomerania (SHIP and SHIP-TREND) covered by the Illumina Infinium HumanExome BeadChip version v1.0 (Exome Chip). Descriptive data (e.g., history of fractures), ultrasonography of the calcaneus, as well as serum concentrations of carboxy-terminal telopeptide of type I collagen, amino-terminal propeptide of type I procollagen, and vitamin D were determined. Comprehensive statistical analyses revealed no association between low-frequency and rare *SLC22A9* variants and bone quality, fractures, and bone turnover markers. Our results indicate that single genetic *SLC22A9* variants do not have a major impact on osteoporosis risk prediction in Europeans, yet findings need to be replicated in larger-scale studies.

## Introduction

Formation and maintenance of bone is a well-balanced process involving osteoclast-mediated bone resorption, osteoblast-mediated bone formation, and remineralization. The peak bone mass is reached around the age of 30; thereafter, bone mass is continuously decreasing because resorption outweighs formation (1). The complex pathophysiology of bone loss is caused by a number of genetic, biochemical, hormonal, and environmental factors (1). In particular, estrogens and their conjugates contribute majorly to maintaining the balance between bone formation and resorption. Accelerated bone loss and compromised bone strength in the elderly increase the risk for osteoporosis affecting hundreds of million people worldwide with major clinical consequences such as fractures, disabilities, and mortality (1–3). It is therefore important to discover markers for the identification of individuals at risk for developing osteoporosis.

In addition to serum biomarkers, multiple genetic variants have been associated with low bone mineral density or increased fracture risk (2, 4–7). Recently, a missense variant in the *SLC22A9* gene was identified in Korean females with osteoporosis but not in control subjects (8). This missense variant (c.T1277C, p.F426S) appears to be unique in Koreans because it has not been found in any population of the public reference exome data sets of the 1000 Genomes Project (9), the Exome Sequencing Project (10), and the Genome Aggregation Database (11) covering in total the genetic variation of more than 141,000 unrelated individuals. *SLC22A9* encodes organic anion transporter 7 (OAT7), which is localized in the hepatocyte basolateral membrane and transports estrone sulfate (12, 13), the major circulating estrogen metabolite considered an important precursor of active estrogen (14). Variants altering transport function, as is the case, e.g., for missense variant c.T1277C (8), may therefore impact on estrone sulfate serum levels and eventually on bone formation. The observation by Ahn et al. (8) has caught our interest because we had systematically characterized OAT7 and identified three genetic variants in Europeans (rs377211288, rs61742518, and rs146027075) leading to functional consequences of estrone sulfate transport in *in vitro* cell systems (13). We therefore hypothesized that genetic variants in *SLC22A9* may be novel biomarkers not only in Koreans but also in Europeans to identify patients at risk for developing osteoporosis. Our aim was to assess the association of *SLC22A9* genetic variants with bone quality, fracture risk and serum bone turnover markers in a cross-sectional population-based cohort study from Germany (SHIP) (15), which had been previously used to identify fracture risk and risk factors for osteoporosis (16).

## Materials and Methods

### Study Population

The study population is based on data from two independent population-based cohorts as described in detail previously: the Study of Health in Pomerania (SHIP) and SHIP-TREND (15, 16). The baseline investigation in SHIP (SHIP-0) took place between 1997 and 2001. The first follow-up (SHIP-1) was conducted 5 years after baseline between 2002 and 2006. The 11-year follow-up investigation (SHIP-2) took place at the same time as SHIP-TREND in 2008–2012. For the present study, we used data from SHIP-1 (*N* = 3,300, 25–88 years), SHIP-2 (*N* = 2,333, 30–93 years), and SHIP-TREND (*N* = 4,420, 20–84 years). All SHIP and SHIP-TREND participants have German citizenship, and the entire sample is Caucasian.

The analyses regarding bone quality and fracture risk were restricted to SHIP-2 and SHIP-TREND (*N* = 6,753), as neither quantitative ultrasound (QUS) measurements at the heel nor details on fracture history were recorded in the other study waves. Among these subjects, 6,067 participants had valid QUS data and were thus eligible for the present study. Subjects with missing data on self-reported fractures, body mass index, or genotyping were excluded. Moreover, we excluded all those who reported intake of antiresorptive drugs used in osteoporosis therapy (bisphosphonates and selective estrogen receptor modulators) as well as glucocorticoids for systemic use, which are well-known causes for secondary osteoporosis. Thus, the study population for the analysis of bone quality and history of fractures included 5,701 participants.

The analyses regarding the bone turnover markers were restricted to SHIP-1 and SHIP-TREND (*N* = 7,720), as both markers were available only in these cohorts. Again, taking into account valid QUS, bone turnover and confounder measurements, as well as medication intake yielded a population of 5,282 subjects for PINP and 5,518 subjects for the respective β-CTX analysis.

Finally, the analyses regarding vitamin D were restricted to the SHIP-TREND cohort (*N* = 4,420). Among these participants, 3,608 had valid QUS, vitamin D, and confounder data, were not taking the above defined medication, and were included in the respective analysis.

Characteristics of the study population are summarized in Table 1. This observational study conformed to strengthening the reporting of observational studies in epidemiology (STROBE) guidelines for observational studies (17). The study followed the recommendations of the Declaration of Helsinki (SHIP-0: version of 1996; SHIP-1, SHIP-2, and SHIP-TREND: version of 2000). The study protocol was approved by the medical ethics committee of the University of Greifswald, and written informed consent was obtained from each study participant.

**Table 1:** Baseline characteristics of the study population.

**Characteristic**	**Male (*N* = 2,771)**	**Female (*N* = 2,930)**
Age (years)	49.9 (15.0)	48.1 (14.5)
BMI (kg/m^2^)	28.6 (4.4)	27.5 (5.5)
Premenopausal (%)	–	1,205 (41.1)
- Intake of oral contraceptives (%)	–	28.9%
Postmenopausal (%)		1,725 (58.9)
- Intake of menopausal hormone replacement therapy (%)	–	6.55%
- Self-reported postmenopausal osteoporosis (%)		9.77%^g^
Fractures (self-reported) (%)	192 (6.9)	223 (7.6)
BUA (dB/MHz)	117.3 (13.8)	109.3 (14.8)
SOS (m/s)	1,564.3 (37.7)	1,562.2 (33.5)
Bone stiffness index	96.6 (18.2)	90.7 (17.6)
High risk for osteoporotic fractures (%)	128 (4.6)	198 (6.8)
PINP (ng/ml)	45.2 (19.0)^a^	46.9 (21.4)^b^
β-CTX (ng/ml)	0.31 (0.18)^c^	0.30 (0.20)^d^
Vitamin D (ng/ml)	24.0 (9.1)^e^	23.8 (9.8)^f^

*Values are means (SD) or n (%)*.

*BMI, body mass index; BUA, broadband ultrasound attenuation; β-CTX, carboxy-terminal telopeptide of type I collagen; PINP, amino-terminal propeptide of type I procollagen; SOS, speed of sound*.

*^a^N = 2,711, ^b^N = 2,571, ^c^N = 2,693, ^d^N = 2,825, ^e^N = 1,785, ^f^N = 1,823; ^g^N = 1,668 postmenopausal women responded to the question regarding a diagnosis of osteoporosis*.

### QUS Measurements

Bone quality was assessed by QUS measurements at the heel. QUS measurements provide information on bone strength and microarchitecture (18) and allow to identify subjects with a high osteoporotic fracture risk (19, 20). The QUS measurements in our study were performed successively on both feet of seated participants by trained and certified examiners using the Achilles InSight device (GE Medical Systems Ultrasound) as described (16). The system measures the speed of sound (SOS) and the broadband ultrasound attenuation (BUA). From these two variables, the stiffness index (SI) was calculated, which is a better indicator for bone quality than its two components alone (21). The individual stiffness index results are compared automatically to values obtained in a normal young reference population (provided with the Achilles InSight). Indices <-2.5 SD indicate a high osteoporotic fracture risk, indices >-2.5 SD and <-1 SD denote a medium osteoporotic fracture risk, and indices >-1 SD indicate a low osteoporotic fracture risk. Data of the foot with the lower stiffness index was used for statistical analyses. Distribution of the stiffness index in pre- and post-menopausal women is shown in Supplementary Figure 1A.

### Fractures

Fractures were self-reported in computer-aided personal interviews. All SHIP-2 and SHIP-TREND participants were asked if they ever had a fracture without a prior accident. Moreover, SHIP-2 participants were asked if they had a fracture after an accident since the baseline examination, while SHIP-TREND participants were asked if they ever had a proximal humerus, hip, femoral, or vertebral fracture after an accident. If any of the questions was answered affirmatively, a positive fracture history was determined.

### Laboratory Measurements

Blood samples were taken from the cubital vein of participants in the supine position. In SHIP-0 and SHIP-1, blood samples were obtained between 8.00 a.m. and 8.00 p.m. from non-fasting participants. In SHIP-2 and SHIP-TREND, blood samples were obtained between 7.00 a.m. and 2.00 p.m. (92.5% of samples between 7.00 and 11.00 a.m.). In SHIP-2, the majority of study participants was non-fasting (92.9%), while in SHIP-TREND, the majority (61.2%) of participants was fasting for at least 8 h. Serum aliquots were stored at −80°C in the Integrated Research Biobank (Liconic, Lichtenstein) of the University Medicine Greifswald and used in accordance with its regulations. Serum intact amino-terminal propeptide of type I procollagen (PINP) and β isomerized carboxy-terminal telopeptides of type I collagen (β-CTX) concentrations, also known as β-CTX concentrations, were determined on the IDS-iSYS Multi-Discipline Automated Analyzer (Immunodiagnostic Systems Limited, Frankfurt am Main, Germany) as described (22). The coefficients of variation for PINP and β-CTX were 7.3 and 12.2% at low concentrations, 5.7 and 10.4% at medium concentrations, and 7.9 and 11.4% at high concentrations of control material in SHIP-1, as well as 4.4 and 7.5% at low concentrations, 4.5 and 5.2% at medium concentrations, and 4.3 and 4.5% at high concentrations of control material in SHIP-TREND. Distribution of the β-CTX concentrations stratified for pre- and post-menopausal women is shown in Supplementary Figure 1B. Serum 25-hydroxy vitamin D was also determined on the IDS-iSYS Multi-Discipline Automated Analyzer as described (23). The coefficients of variation for vitamin D were 17.2% at low concentrations, 9.5% at medium concentrations, and 8.5% at high concentrations of control material in SHIP-TREND.

### Genotypes

SHIP and SHIP-TREND samples were genotyped using the Illumina Infinium HumanExome BeadChip version v1.0 (Exome Chip). Genomic DNA was hybridized according to the manufacturer's standard recommendations at the Helmholtz Zentrum München. Genotypes were determined using the Genotyping Module v1.9.4 from the GenomeStudio 2011.11 software with the HumanExome-12-v1-0-B manifest file and the CHARGE_ExomeChip_v1.0 cluster file. Standard quality control steps were carried out using PLINK v1.90 (24, 25). Contaminated samples and samples with a call rate of <90%, extreme heterozygosity, extensive estimated identical-by-descent sharing with a large number of samples, or gender mismatch were excluded. Population outliers were identified by principal component analysis with smartpca version 10210 using the Exome Chip ancestry information marker. The final dataset included 8,219 individuals (3,949 SHIP and 4,270 SHIP-TREND) with an average call rate of 99.98%. In addition, rs377211288 was genotyped using KASP™ technology (LGC Genomics Ltd., Hoddesdon, UK) since this variant was not covered by the Exome Chip. Primer sequences were as follows: forward primer allele_X: ATGAGGCCAGAGAAGTGTCGTC, forward primer allele_Y: CATGAGGCCAGAGAAGTGTCGTT, common reverse primer: GAGCTGCCACTGAGGATGAACAAA.

### Statistical Analysis

Analyses on European ancestry were performed by using the software packages PLINK v1.90 and R (version 3.4.0 2017-04-21) (24, 25). Hardy–Weinberg equilibrium calculations were used to compare observed and expected allele and genotype frequencies within the study population. Single-variant association analyses on all variants were performed in PLINK v1.90 considering three genetic models: additive (homozygote allele 1 vs. heterozygote vs. homozygote allele 2), dominant (homozygote allele 1 vs. heterozygote + homozygote allele 2), and recessive (homozygote allele 1 + heterozygote vs. homozygote allele 2). Multiple linear regression analysis was performed for the traits bone quality, vitamin D, β-CTX, and PINP and multiple logistic regression analysis for fractures. Models were adjusted for age at blood taking, sex, BMI, study cohort (except for vitamin D), and the first 10 principal components to correct for population stratification. In case of the sex-stratified analyses for women, models were additionally adjusted for menopause (except for vitamin D). Traits for PINP and β-CTX were log-transformed and winsorized by 5 standard deviations from the mean to reduce the impact of outliers. For vitamin D, a rank-based inverse normal transformation was performed. Bonferroni correction was used to adjust *P*-values for multiple testing. A *P* < 0.05 was considered significant.

## Results

### *SLC22A9* Genetic Variants and Allele Frequencies

A total of 24 missense variants in the *SLC22A9* gene were analyzed (Table 2). Twenty-three variants were assessed using the Exome Chip, and from these, variant exm920540 was excluded due to an insufficient call rate. Variant rs377211288 was genotyped by KASP™ technology because it is not covered by the Exome Chip. From the 23 genotyped variants, only 14 variants were detected. The 2 variants rs61742518 (p.T433M) and rs146027075 (p.I479M) occurred with minor allele frequencies of 2.68 and 0.96%, respectively, and were therefore the only low-frequency variants. The remaining 12 variants occurred only heterozygously and were rare variants with minor allele frequencies between 0.1 and 0.01% (5 variants) or occurring in single individuals (7 “singleton” variants). The genotype distributions of all variants fitted to the Hardy–Weinberg equilibrium and are given in Table 3. Compared to a large exome–genome–NGS cohort of non-Finnish Europeans (gnomAD including 56,000 samples) (11), all variants were covered with comparable minor allele frequencies (Table 2).

**Table 2:** Minor allele frequencies (MAF) and other annotations of the *SLC22A9* genetic variants assessed by the Illumina ExomeChip array or KASP™ sequencing in the SHIP and SHIP-TREND study cohort in comparison to the data for non-Finnish Europeans from the Genome Aggregation Database (gnomAD).

**refSNP_ID**	**Exome_ID**	**db SNP alleles**	**Contig position^**a**^**	**Minor allele**	**Amino acid change**	**MAF (%) This study**	**MAF (%) gnomAD^**b**^**
rs139996395	exm920540	A/G	63137767	G	p.Asp80Gly	Excluded^c^	0.0566
rs377211288^d^		C/T	63137796	T	p.Arg90Cys	0.0451	0.0116
rs200727459	exm920544	G/A	63137797	A	p.Arg90His	0	0.0116
rs138297035	exm920554	G/A	63138694	A	p.Gly164Ser	0.0088	0.0031
rs202164269	exm920556	C/G	63141131	G	p.Phe174Leu	0.0877	0.0741
rs180967669	exm1726437	G/C	63141180	C	p.Ala191Pro	0	7.8*e*-4
rs139591412	exm920572	C/T	63141435	T	p.Ala244Val	0.0088	0.0156
rs79899382	exm920573	A/T	63141443	T	p.Thr247Ser	0	0
rs3737458	exm920575	G/A	63141471	A	p.Arg256Gln	0.0088	0.0381
rs201479912	exm920579	G/A	63143137	A	p.Arg284Gln	0	0.0026
rs200498139	exm920580	C/A	63143153	A	p.Gln289Lys	0.0088	0.0163
rs1801401	exm920582	C/A	63143227	A	p.Thr314Asn	0	0.0062
rs201804022	exm920585	C/A	63149670	A	p.Gln332Lys	0	0.0236
rs141060614	exm920591	C/T	63149746	T	p.Thr357Met	0.0088	0.0048
rs182247457	exm920593	A/G	63173982	G	p.Met363Val	0.0702	0.0853
rs138875094	exm920594	T/G	63174033	G	p.Phe380Val	0	0.0023
rs78019460	exm920597	T/C	63174102	C	p.Tyr403His	0	0
rs61742518	exm920601	C/T	63175593	T	p.Thr433Met	2.6837	2.979
rs144303933	exm920603	G/A	63175599	A	p.Arg435His	0.0088	0.0506
rs139254772	exm920609	T/A	63175663	A	p.His456Gln	0.0263	0.0148
rs147323107	exm920610	C/A	63175679	A	p.Pro462Thr	0	0.0016
rs146027075	exm920613	A/G	63176187	G	p.Ile479Met	0.9560	0.8855
rs149660130	exm920619	T/G	63176294	G	p.Leu515Arg	0.0175	0.0155
rs142543443	exm920628	C/T	63177324	T	p.Thr551Met	0.0088	0.0124

a *Contig position refers to chromosome 11, GRCh37 build*.

b *Values from gnomAD v2.1.1 for non-Finnish Europeans (https://gnomad.broadinstitute.org) (11)*.

c *Variant excluded due to insufficient call rate*.

d *Variant sequenced by KASP™ technology since it was not included on the ExomeChip*.

**Table 3:** Genotype distributions for the 14 *SLC22A9* genetic variants identified in the SHIP and SHIP-TREND study cohort.

**refSNP_ID**	**db SNP alleles**	**Genotype**	**Number of individuals^**a**^**	**Observed heterozygote frequency**	**Expected heterozygote frequency**	**HWE exact *p*-value**
rs377211288 (NA)	C/T	CC	5, 543	0.0009012	0.0009008	1
		CT	5			
		TT	0			
rs138297035 (exm920554)	G/A	GG	5, 700	0.0001754	0.0001754	1
		GA	1			
		AA	0			
rs202164269 (exm920556)	C/G	CC	5, 691	0.001754	0.001753	1
		CG	10			
		GG	0			
rs139591412 (exm920572)	C/T	CC	5, 639	0.000173	0.0001773	1
		CT	1			
		TT	0			
rs3737458 (exm920575)	G/A	GG	5, 700	0.0001754	0.0001754	1
		GA	1			
		AA	0			
rs200498139 (exm920580)	C/A	CC	5, 700	0.0001754	0.0001754	1
		CA	1			
		AA	0			
rs141060614 (exm920591)	C/T	CC	5, 700	0.0001754	0.0001754	1
		CT	1			
		TT	0			
rs182247457 (exm920593)	A/G	AA	5, 693	0.001403	0.001402	1
		AG	8			
		GG	0			
rs61742518 (exm920601)	C/T	CC	5, 397	0.05297	0.05223	0.4424
		CT	302			
		TT	2			
rs144303933 (exm920603)	G/A	GG	5, 700	0.0001754	0.0001754	1
		GA	1			
		AA	0			
rs139254772 (exm920609)	T/A	TT	5, 698	0.0005262	0.0005261	1
		TA	3			
		AA	0			
rs146027075 (exm920613)	A/G	AA	5, 593	0.01877	0.01894	0.4062
		AG	107			
		GG	1			
rs149660130 (exm920619)	T/G	TT	5, 699	0.0003508	0.0003508	1
		TG	2			
		GG	0			
rs142543443 (exm920628)	C/T	CC	5, 700	0.0001754	0.0001754	1
		CT	1			
		TT	0			

a *Numbers are for bone quality*.

*HWE, Hardy–Weinberg equilibrium*.

### Associations of *SLC22A9* Genetic Variants With Bone Quality, Fracture Risk, and Serum Bone Turnover Markers

**Figure 1:**
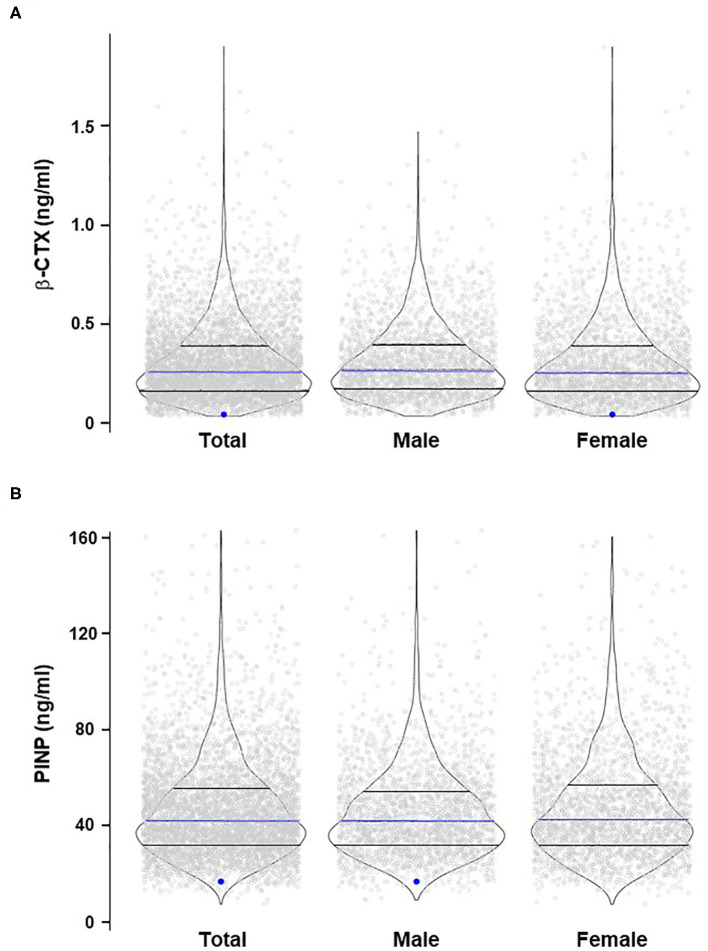
Violin plots of β-CTX **(A)** and PINP **(B)** distribution in the study cohort stratified for sex. The blue dots indicate the heterozygous samples with significant associations between rs200498139 and β-CTX **(A)** and rs3737458 and PINP **(B)**. The blue lines indicate the median values; lower and upper black lines indicate the 1st and 3rd quartiles, respectively.

Comprehensive single-variant association analyses were performed for all 14 *SLC22A9* genetic variants considering an additive genetic model. For variants rs61742518 and rs146027075, a dominant and recessive genetic model was additionally considered because individuals with the respective homozygous variant alleles were identified. After correction for multiple testing, there were no significant associations between QUS-based stiffness index, fractures, and serum bone turnover markers and any of the low-frequency and 10 rare variants regardless of whether all individuals were analyzed together or whether individuals were stratified by sex (Supplementary Table 1). The singleton variants rs200498139 and rs3737458 were significantly associated with β-CTX and PINP, respectively, after correcting for multiple testing (Supplementary Tables 1D,E). The two heterozygous individuals carrying either variant had very low β-CTX or PINP serum levels (Figure 1).

## Discussion

Estrogens play an important role in the resorption and formation of bone and their age-dependent decrease is a major factor for increased bone fragility and fragility-related fractures, particularly in postmenopausal women (1). The metabolite estrone sulfate is considered a major precursor of active estrogen with serum levels about 10-fold higher than unconjugated estrone (14). The absorption, disposition, and elimination of estrone sulfate depend on multiple membrane transporters from several families, including uptake transporters, e.g., the organic anion transporters OAT3 (SLC22A8) and OAT4 (SLC22A11), the organic anion transporting polypeptides OATP1B1 (SLCO1B1), OATP1B3 (SLCO1B3), and OATP2B1 (SLCO2B1), as well as ATP-dependent efflux transporters of the C subfamily (ABCC1, ABCC2, ABCC3, and ABCC11) and ABCG2 (26–29). More recent studies have identified OAT7 as an additional estrone sulfate transporter (12, 13). A study examining Korean females (8) even found the reduced-function variant p.F426S of OAT7 in women with osteoporosis but not in control subjects. This study (8) failed, however, in providing exact methods regarding osteoporosis diagnosis.

In the course of a systematic characterization, we had identified and characterized three functionally relevant OAT7 variants in Europeans: p.R90C (rs377211288), p.T433M (rs61742518), and p.I479M (rs146027075) (13). We therefore tested the hypothesis whether these variants as well as additional missense variants assessed by the Exome Chip also impact on bone quality, assessed by heel QUS, in a population-based European cohort. As there was no bone mineral density measurement in our cohort, results are limited to provide indices of the osteoporotic fracture risk instead of osteoporosis itself. Neither the two low-frequency variants nor the five rare nor most singleton variants were associated with the QUS-based osteoporotic fracture risk, self-reported fractures, or serum bone turnover markers. The observed significant association of two singleton variants with β-CTX and PINP is probably a spurious association and will need confirmation in a much larger cohort. A major reason for this lack of association may be the complex pathophysiology of bone loss, which is not only caused by a number of genetic, biochemical, hormonal, and environmental factors but apparently also depends, among others, on ethnicity and lifestyle (1, 30). Moreover, it appears that variants in a multitude of genes are involved in maintenance of bone, each having only a small effect size (1, 6, 7, 31). Morris et al., for example, identified four low-frequency variants with small effect sizes on heel BMD mapping to genes *SLC22A24, SLC22A25*, and *SLC22A10*, being close to the *SLC22A9* gene but not to the *SLC22A9* gene itself (7). However, these variants (rs117269586, rs138722111, rs35740247, and rs56260618), which are available as imputed variants in the SHIP and SHIP-TREND cohorts (32), were not significantly associated with the bone stiffness index. Yet, a role of this gene region in bone biology cannot be ruled out and needs to be analyzed in studies using cohorts of diagnosed osteoporosis patients, particularly because *SLC22A24* had been recently identified as an additional transporter of estrone sulfate (33).

We studied a population-based cohort drawn from a general population of Europeans that has been previously used to identify risk factors of osteoporosis (16). This population-based study design and the number of participants are the main strengths of the present study. While our study provides reliable results on bone turnover and the osteoporotic fracture risk, evidence on osteoporosis itself cannot be provided as dual-energy X-ray absorptiometry (DXA) scans, using ionizing radiation, were impossible in our population-based setting due to ethical objections. However, despite the large number of participants, this study has the limitation that it still may not be large enough to estimate the effect of rare variants with minor allele frequencies <0.01% and might be underpowered to identify significant associations. This is particularly important for *SLC22A9*, for which only rare variants exist in Europeans, except for one low-frequency and one rare variant. Moreover, it cannot be excluded that rare variants of *SLC22A9*, not captured by the Exome Chip, with large effect sizes may exist, which needs investigation in a much larger cohort. Novel technologies such as whole-genome and whole-exome sequencing (31) may be instrumental in identifying novel rare variants in *SLC22A9* associated with bone density.

In conclusion, we found no association of *SLC22A9* missense variants with QUS-based bone quality, fractures, and serum bone turnover markers. Our results indicate that, in contrast to the situation in Koreans, single *SLC22A9* genetic variants apparently do not have a major impact on osteoporosis risk prediction in Europeans.

## Data Availability Statement

The datasets presented in this study can be found in online repositories. The names of the repository/repositories and accession number(s) can be found in the article/Supplementary Material.

## Ethics Statement

SHIP data are publicly available for scientific and quality control purposes. The informed consent obtained from the participants of the SHIP studies does not cover data storage in public databases due to confidentially reasons. Data usage can be applied for via https://www.fvcm.med.uni-greifswald.de/dd_service/data_use_intro.php?lang=ger, an interface provided by the host institute of the SHIP study to ensure compliance with all legislation. The website is in German language only but several documents, including the application form concerning delivery and use of data and/or sample material are available in English https://www.fvcm.med.uni-greifswald.de/Web/Anlage_1_NutzungsO_(Antrag_Daten-Probennutzung)_EN.pdf. The staff of the Transferstelle (transfer@uni-greifswald.de) will on request, detail the restrictions and any conditions under which access to the data may be provided and support with the application for the data.

## Author Contributions

AN, ES, HW, and MS designed the study. AH, UV, and HW conducted the study and collected data. SW and AN analyzed data. AN, ES, and MS interpreted data. AN, AH, SW, and MS drafted the manuscript. All authors revised the manuscript content and approved the final version.

## Conflict of Interest

The authors declare that the research was conducted in the absence of any commercial or financial relationships that could be construed as a potential conflict of interest.
