# Supplementary material for: The Membrane Transporter OAT7 (SLC22A9) Is Not a Susceptibility Factor for Osteoporosis in Europeans
Source: Front Endocrinol (Lausanne). 2020 Aug 18;11:532. doi: 10.3389/fendo.2020.00532 (PMC7461822; doi:10.3389/fendo.2020.00532)
Supplement: Supplementary file 1 [file Data_Sheet_1.docx]

**Supplementary Figure 1.** Violin plots of the stiffness index (A) and β-CTX (B) distribution in the study cohort stratified for pre- and post-menopause. The blue lines indicate the median values, lower and upper dotted lines indicate the 1st and 3rd quartiles, respectively. The red diamond indicates the mean. As expected, the β-CTX values were higher and the stiffness index was lower in post-menopausal women than in pre-menopausal women.

**Supplementary Table 1.** Analysis of associations between the 14 *SLC22A9* genetic variants identified in the SHIP and SHIP-TREND study cohort with bone quality, fractures, and bone turnover markers

**A Bone quality**

| **refSNP_ID (ExomeID)** | **Genetic model** | | **N individuals** | | | **Bone quality mean (sd)** | | | **Effect estimate** | **Standard error** | **unadj. p-value** | **adj. p-value (BONF)** | **adj. p-value (FDR BH)** |
| --- | --- | --- | --- | --- | --- | --- | --- | --- | --- | --- | --- | --- | --- |
|  |  |  | **AA** | **AB** | **BB** | **AA** | **AB** | **BB** |  |  |  |  |  |
| rs377211288  (-) | Additive | CC vs. CT | 5543 | 5 | 0 | 93.52  (18.08) | 103.82  (23.12) | - | 6.454 | 7.583 | 0.3948 | 0.3948 | 0.3948 |
| rs138297035  (exm920554) | Additive | GG vs. GA | 5700 | 1 | 0 | 93.53  (18.13) | 109.16  (-) | - | 17.49 | 17.00 | 0.3035 | 1 | 0.9904 |
| rs202164269  (exm920556) | Additive | CC vs. CG | 5691 | 10 | 0 | 93.52  (18.13) | 101.95  (16.26) | - | 3.346 | 5.376 | 0.5337 | 1 | 0.9904 |
| rs139591412  (exm920572) | Additive | CC vs. CT | 5639 | 1 | 0 | 93.58  (18.13) | 128.97  (-) | - | 37.48 | 16.998 | 0.0275 | 0.3572 | 0.3572 |
| rs3737458  (exm920575) | Additive | GG vs. GA | 5700 | 1 | 0 | 93.53  (18.12) | 100.43  (-) | - | 1.73 | 16.961 | 0.9188 | 1 | 0.9904 |
| rs200498139  (exm920580) | Additive | CC vs. CA | 5700 | 1 | 0 | 93.53  (18.13) | 107.65  (-) | - | 13.54 | 16.961 | 0.4248 | 1 | 0.9904 |
| rs141060614  (exm920591) | Additive | CC vs. CT | 5700 | 1 | 0 | 93.53  (18.13) | 77.14  (-) | - | -8.738 | 16.964 | 0.6065 | 1 | 0.9904 |
| rs182247457  (exm920593) | Additive | AA vs. AG | 5693 | 8 | 0 | 93.53  (18.11) | 95.45  (26.99) | - | 4.497 | 6.004 | 0.4539 | 1 | 0.9904 |
| rs61742518  (exm920601) | Additive | CC vs. CT vs. TT | 5397 | 302 | 2 | 93.56  (18.10) | 93.09  (18.64) | 71.98  (7.79) | -0.392 | 0.991 | 0.6926 | 1 | 0.9904 |
|  | Dominant | CC vs. CT/TT | 5397 | 302 | 2 |  |  |  | -0.288 | 1.001 | 0.7733 | 1 | 0.9904 |
|  | Recessive | CC/CT vs. TT | 5397 | 302 | 2 |  |  |  | -16.06 | 12.012 | 0.1814 | 0.3627 | 0.3627 |
| rs144303933  (exm920603) | Additive | GG vs. GA | 5700 | 1 | 0 | 93.53  (18.13) | 79.21  (-) | - | -5.722 | 16.969 | 0.7360 | 1 | 0.9904 |
| rs139254772  (exm920609) | Additive | TT vs. TA | 5698 | 3 | 0 | 93.52  (18.12) | 108.79  (24.97) | - | 16.18 | 9.8 | 0.0987 | 1 | 0.6416 |
| rs146027075  (exm920613) | Additive | AA vs. AG vs. GG | 5593 | 107 | 1 | 93.55  (18.11) | 92.66  (19.34) | 94.67  (-) | -0.32 | 1.627 | 0.8442 | 1 | 0.9904 |
|  | Dominant | AA vs. AG/GG | 5593 | 107 | 1 |  |  |  | -0.239 | 1.65 | 0.8850 | 1 | 0.9904 |
|  | Recessive | AA/AG vs. GG | 5593 | 107 | 1 |  |  |  | -9.536 | 16.971 | 0.5742 | 1 | 0.5742 |
| rs149660130  (exm920619) | Additive | TT vs. TG | 5699 | 2 | 0 | 93.53  (18.13) | 93.74  (10.78) | - | -0.145 | 12.003 | 0.9904 | 1 | 0.9904 |
| rs142543443  (exm920628) | Additive | CC vs. CT | 5700 | 1 | 0 | 93.53  (18.13) | 86.06  (-) | - | 0.587 | 16.964 | 0.9724 | 1 | 0.9904 |

**B Fractures**

| **refSNP_ID (ExomeID)** | **Genetic model** | | **N individuals** | | | **Cases** | | | **Controls** | | | **Effect estimate** | **Standard error** | **unadj. p-value** | **adj. p-value (BONF)** | **adj. p-value (FDR BH)** |
| --- | --- | --- | --- | --- | --- | --- | --- | --- | --- | --- | --- | --- | --- | --- | --- | --- |
|  |  |  | **AA** | **AB** | **BB** | **AA** | **AB** | **BB** | **AA** | **AB** | **BB** |  |  |  |  |  |
| rs182247457  (exm920593) | Additive | AA vs. AG | 5693 | 8 | 0 | 414 | 1 | 0 | 5279 | 7 | 0 | 0.430 | 1.08 | 0.6904 | 1 | 0.6904 |
| rs61742518  (exm920601) | Additive | CC vs. CT vs. TT | 5397 | 302 | 2 | 396 | 19 | 0 | 5001 | 283 | 2 | -0.1752 | 0.2421 | 0.4693 | 1 | 0.6891 |
|  | Dominant | CC vs. CT/TT | 5397 | 302 | 2 | 396 | 19 | 0 | 5001 | 283 | 2 | -0.1691 | 0.2438 | 0.4879 | 1 | 0.6904 |
| rs146027075  (exm920613) | Additive | AA vs. AG vs. GG | 5593 | 107 | 1 | 409 | 6 | 0 | 5184 | 101 | 1 | -0.2744 | 0.4233 | 0.5168 | 1 | 0.6891 |
|  | Dominant | AA vs. AG/GG |  |  |  |  |  |  |  |  |  | -0.2715 | 0.4253 | 0.5232 | 1 | 0.6904 |
| rs149660130  (exm920619) | Additive | TT vs. TG | 5699 | 2 | 0 | 414 | 1 | 0 | 5285 | 1 | 0 | 2.3580 | 1.4274 | 0.0985 | 0.3940 | 0.3940 |

**C Vitamin D**

| **refSNP_ID (ExomeID)** | **Genetic model** | | **N individuals** | | | **Vitamin D mean (sd)** | | | **Effect estimate** | **Standard error** | **unadj. p-value** | **adj. p-value (BONF)** | **adj. p-value (FDR BH)** |
| --- | --- | --- | --- | --- | --- | --- | --- | --- | --- | --- | --- | --- | --- |
|  |  |  | **AA** | **AB** | **BB** | **AA** | **AB** | **BB** |  |  |  |  |  |
| rs377211288  (-) | Additive | CC vs. CT | 3550 | 3 | 0 | 23.90  (9.47) | 20.93  (3.42) | - | -0.214 | 0.575 | 0.7104 | 0.7104 | 0.7104 |
| rs202164269  (exm920556) | Additive | CC vs. CG | 3602 | 6 | 0 | 23.89  (9.47) | 27.72  (10.03) | - | 0.463 | 0.406 | 0.2548 | 1 | 0.4530 |
| rs139591412  (exm920572) | Additive | CC vs. CT | 3573 | 1 | 0 | 23.89  (9.49) | 40.7  (-) | - | 1.424 | 0.996 | 0.1529 | 0.9177 | 0.4530 |
| rs182247457  (exm920593) | Additive | AA vs. AG | 3605 | 3 | 0 | 23.89  (9.46) | 37.07  (13.91) | - | 1.129 | 0.574 | 0.0493 | 0.2956 | 0.2956 |
| rs61742518  (exm920601) | Additive | CC vs. CT vs. TT | 3424 | 183 | 1 | 23.93  (9.52) | 23.40  (8.55) | 23.4  (-) | -0.041 | 0.075 | 0.5820 | 1 | 0.6984 |
|  | Dominant | CC vs. CT/TT | 3424 | 183 | 1 |  |  |  | -0.042 | 0.075 | 0.5786 | 1 | 0.6943 |
|  | Recessive | CC/CT vs. TT | 3424 | 183 | 1 |  |  |  | 0.007 | 0.996 | 0.9946 | 1 | 0.9946 |
| rs139254772  (exm920609) | Additive | TT vs. TA | 3607 | 1 | 0 | 23.90  (9.47) | 23.8  (-) | - | -0.012 | 0.994 | 0.9907 | 1 | 0.9907 |
| rs146027075  (exm920613) | Additive | AA vs. AG vs. GG | 3531 | 76 | 1 | 23.87  (9.43) | 25.64  (11.18) | 16.9  (-) | 0.116 | 0.112 | 0.3020 | 1 | 0.4530 |
|  | Dominant | AA vs. AG/GG | 3531 | 76 | 1 |  |  |  | 0.132 | 0.115 | 0.2477 | 1 | 0.3822 |
|  | Recessive | AA/AG vs. GG | 3531 | 76 | 1 |  |  |  | -0.894 | 0.994 | 0.3685 | 0.7370 | 0.7370 |

**D Carboxy-terminal telopeptide of type I collagen (βCTX)**

| **refSNP_ID (ExomeID)** | **Genetic model** | | **N individuals** | | | **CTX mean (sd)** | | | **Effect estimate** | **Standard error** | **unadj. p-value** | **adj. p-value (BONF)** | **adj. p-value (FDR BH)** |
| --- | --- | --- | --- | --- | --- | --- | --- | --- | --- | --- | --- | --- | --- |
|  |  |  | **AA** | **AB** | **BB** | **AA** | **AB** | **BB** |  |  |  |  |  |
| rs377211288 (-) | Additive | CC vs. CT | 5367 | 5 | 0 | 0.302  (0.194) | 0.372  (0.149) | - | 0.268 | 0.283 | 0.3440 | 0.3440 | 0.3440 |
| rs138297035  (exm920554) | Additive | GG vs. GA | 5517 | 1 | 0 | 0.302  (0.194) | 0.271  (-) | - | -0.066 | 0.634 | 0.9170 | 1 | 0.9552 |
| rs202164269 (exm920556) | Additive | CC vs. CG | 5508 | 10 | 0 | 0.302  (0.194) | 0.241  (0.123) | - | -0.164 | 0.200 | 0.4145 | 1 | 0.7106 |
| rs139591412 (exm920572) | Additive | CC vs. CT | 5458 | 1 | 0 | 0.302  (0.193) | 0.189  (-) | - | -0.37 | 0.634 | 0.5596 | 1 | 0.7462 |
| rs3737458 (exm920575) | Additive | GG vs. GA | 5517 | 1 | 0 | 0.302  (0.194) | 0.205  (-) | - | -0.407 | 0.633 | 0.5200 | 1 | 0.7462 |
| rs200498139 (exm920580) | Additive | CC vs. CA | 5517 | 1 | 0 | 0.302  (0.194) | 0.041  (-) | - | -1.929 | 0.632 | 0.0023 | 0.0275 | 0.0275 |
| rs141060614 (exm920591) | Additive | CC vs. CT | 5517 | 1 | 0 | 0.302  (0.194) | 0.29  (-) | - | 0.137 | 0.633 | 0.8287 | 1 | 0.9552 |
| rs182247457 (exm920593) | Additive | AA vs. AG | 5510 | 8 | 0 | 0.302  (0.194) | 0.361  (0.188) | - | 0.197 | 0.224 | 0.3789 | 1 | 0.7106 |
| rs61742518 (exm920601) | Additive | CC vs. CT vs. TT | 5227 | 289 | 2 | 0.302  (0.195) | 0.295  (0.177) | 0.542  (0.042) | -0.002 | 0.038 | 0.9552 | 1 | 0.9552 |
|  | Dominant | CC vs. CT/TT | 5227 | 289 | 2 |  |  |  | -0.008 | 0.038 | 0.8436 | 1 | 0.9170 |
|  | Recessive | CC/CT vs. TT | 5227 | 289 | 2 |  |  |  | 0.74 | 0.448 | 0.0988 | 0.1975 | 0.1975 |
| rs144303933 (exm920603) | Additive | GG vs. GA | 5517 | 1 | 0 | 0.302  (0.194) | 0.627  (-) | - | 1.068 | 0.633 | 0.0915 | 1 | 0.3661 |
| rs139254772 (exm920609) | Additive | TT vs. TA | 5515 | 3 | 0 | 0.302  (0.194) | 0.203  (0.055) | - | -0.345 | 0.366 | 0.3452 | 1 | 0.7106 |
| rs146027075 (exm920613) | Additive | AA vs. AG vs. GG | 5411 | 106 | 1 | 0.301  (0.192) | 0.335  (0.245) | 0.639  (-) | 0.111 | 0.061 | 0.0687 | 0.8245 | 0.3661 |
|  | Dominant | AA vs. AG/GG | 5411 | 106 | 1 |  |  |  | 0.107 | 0.062 | 0.0842 | 1 | 0.3661 |
|  | Recessive | AA/AG vs. GG | 5411 | 106 | 1 |  |  |  | 0.771 | 0.633 | 0.2233 | 0.4465 | 0.2233 |
| rs142543443 (exm920628) | Additive | CC vs. CT | 5517 | 1 | 0 | 0.302  (0.194) | 0.509  (-) | - | 0.646 | 0.633 | 0.3071 | 1 | 0.7106 |

**E Amino-terminal propeptide of type I procollagen (PINP)**

| **refSNP_ID (ExomeID)** | **Genetic model** | | **N individuals** | | | **PINP mean (sd)** | | | **Effect estimate** | **Standard error** | **unadj. p-value** | **adj. p-value (BONF)** | **adj. p-value (FDR BH)** |
| --- | --- | --- | --- | --- | --- | --- | --- | --- | --- | --- | --- | --- | --- |
|  |  |  | **AA** | **AB** | **BB** | **AA** | **AB** | **BB** |  |  |  |  |  |
| rs377211288 (-) | Additive | CC vs. CT | 5414 | 5 | 0 | 46.14 (20.25) | 58.32  (11.45) | - | 0.27 | 0.183 | 0.1397 | 0.1397 | 0.1397 |
| rs138297035 (exm920554) | Additive | GG vs. GA | 5567 | 1 | 0 | 46.087  (20.25) | 40.2  (-) | - | -0.061 | 0.409 | 0.8811 | 1 | 0.8811 |
| rs202164269 (exm920556) | Additive | CC vs. CG | 5558 | 10 | 0 | 46.09  (20.25) | 43.74  (17.97) | - | -0.029 | 0.129 | 0.8258 | 1 | 0.8811 |
| rs139591412 (exm920572) | Additive | CC vs. CT | 5507 | 1 | 0 | 46.053  (20.28) | 32.5  (-) | - | -0.304 | 0.409 | 0.4567 | 1 | 0.7440 |
| rs3737458 (exm920575) | Additive | GG vs. GA | 5567 | 1 | 0 | 46.09  (20.24) | 16.8  (-) |  | -0.929 | 0.408 | 0.0228 | 0.2738 | 0.2738 |
| rs200498139 (exm920580) | Additive | CC vs. CA | 5567 | 1 | 0 | 46.09  (20.25) | 26.8  (-) |  | -0.458 | 0.408 | 0.2621 | 1 | 0.7440 |
| rs141060614 (exm920591) | Additive | CC vs. CT | 5567 | 1 | 0 | 46.08  (20.25) | 59.3  (-) |  | 0.278 | 0.48 | 0.4960 | 1 | 0.7440 |
| rs182247457 (exm920593) | Additive | AA vs. AG | 5560 | 8 | 0 | 46.09  (20.25) | 43.16  (17.12) | - | -0.029 | 0.144 | 0.8415 | 1 | 0.8811 |
| rs61742518 (exm920601) | Additive | CC vs. CT vs. TT | 5273 | 293 | 2 | 46.09  (20.32) | 45.71  (18.55) | 88.50  (19.94) | 0.014 | 0.024 | 0.5614 | 1 | 0.7486 |
|  | Dominant | CC vs. CT/TT | 5273 | 293 | 2 |  |  |  | 0.009 | 0.024 | 0.7092 | 1 | 0.8811 |
|  | Recessive | CC/CT vs. TT | 5273 | 293 | 2 |  |  |  | 0.731 | 0.289 | 0.0114 | 0.0228 | 0.0228 |
| rs144303933 (exm920603) | Additive | GG vs. GA | 5567 | 1 | 0 | 46.08  (20.25) | 56.4  (-) | - | 0.506 | 0.408 | 0.2151 | 1 | 0.7440 |
| rs139254772 (exm920609) | Additive | TT vs. TA | 5565 | 3 | 0 | 46.09  (20.25) | 34.7  (3.72) | - | -0.192 | 0.236 | 0.4148 | 1 | 0.7440 |
| rs146027075 (exm920613) | Additive | AA vs. AG vs. GG | 5460 | 107 | 1 | 46.04  (20.18) | 47.91  (23.07) | 79.8  (-) | 0.033 | 0.039 | 0.3959 | 1 | 0.7440 |
|  | Dominant | AA vs. AG/GG | 5460 | 107 | 1 |  |  |  | 0.03 | 0.04 | 0.4538 | 1 | 0.7440 |
|  | Recessive | AA/AG vs. GG | 5460 | 107 | 1 |  |  |  | 0.469 | 0.408 | 0.2502 | 0.5003 | 0.2502 |
| rs142543443 (exm920628) | Additive | CC vs. CT | 5567 | 1 | 0 | 46.08  (20.25) | 57.2  (-) | - | 0.379 | 0.408 | 0.3527 | 1 | 0.7440 |

BONF, Bonferroni correction; FDR BH, False-discovery rate according to Benjamini-Hochberg
